# Prevalence of peripheral arterial disease and related risk factors in Turkish elders

**DOI:** 10.1186/1471-2296-12-96

**Published:** 2011-09-19

**Authors:** Nil Tekin, Muammer Baskan, Teoman Yesilkayali, Ozalp Karabay

**Affiliations:** 1 Narlidere Geriatric Care Center and Residential Home, Narlidere, Izmir, Turkey; 2 Department of Cardiovascular Surgery, Dokuz Eylul University Hospital, Balcova, Izmir, Turkey

## Abstract

**Background:**

It is known that prevalence of peripheral arterial disease being a widespread atherosclerotic vascular disease increases by age. On the other hand, no comprehensive study showing the prevalence of peripheral arterial disease in Turkish elders is seen. In this study, it is aimed to assess prevalence of peripheral arterial disease and related risk factors in Turkish elders in primary health center.

**Methods:**

507 elderly staying at Narlidere Geriatric Care Center and Residential Home and accepting to participate in the study were included in the study. Epidemiological data for diagnosis of peripheral arterial disease, risk factors, findings of physical examination and ankle brachial index measurements were assessed in the study. Data were analyzed in terms of prevalence of peripheral arterial disease, age and gender relation and other cardiovascular risk factors.

**Results:**

Of the participants, 317 (62.5%) were female. The mean age was 77.61 ± 6.93 years (62-102). The most wide-spread chronic diseases in elderly included hypertension, coronary artery disease, hyperlipidemia and Type 2 DM, respectively. On the other hand, only 7 (1.4%) elderly were diagnosed with peripheral arterial disease. The number of elderly ABI of whom was measured as < 0.90 and sent to cardiovascular surgery polyclinic with the diagnosis of peripheral arterial disease was assessed as 30 (5.9%). Intermittent claudication was seen in about half of these patients.

**Conclusions:**

Peripheral arterial disease is expected to be seen prevailing in elderly. However, it was determined at very low rate before the study due to the fact that the disease cannot be diagnosed clinically especially in early-period. Peripheral arterial disease determined in the study is lower than expected as per the age group. This can be associated with practices of geriatrics nursing and family practice including continuous care to reduce cardiovascular risk factors of patients staying at the unit.

## Background

Cardiovascular diseases, primarily coronary artery disease (CAD), are the most frequent causes of death in Turkey as well as many other European countries [1,2]. Major cardiovascular events and stroke also occur in individuals not thought to have cardiovascular disease. Smoking, hypertension, hyperlipidemia and diabetes mellitus are the main risk factors for cardiovascular disease [3,4]. However, one high risk factor that is often ignored is peripheral arterial disease (PAD) [4].

PAD is an atherosclerotic vascular disease that affects about 8-10 million people in the USA [5]. Its prevalence increases with age. Assessments using non-invasive methods suggest that PAD affects 3% of individuals in the general population below 60 years of age, over 8% of those aged 60-69 years and up to 20% of individuals aged 70 years and above [5-10].

Although the prevalence of PAD in Turkey is thought to be similar, it has not been studied extensively, especially in elderly individuals. PAD can include a set of disorders that cause progressive stenosis, occlusion of arteries other than intracranial vessels and coronary arterial branches of the aorta and aneurysm dilatations. However, the term PAD is usually used to indicate disorders in circulation in the lower extremities [5,10-14].

Several clinical markers have been associated with primary cardiovascular diseases and related risk factors. In addition, simple, non-invasive and cost-efficient tests are very useful for determining vascular risks [10]. One of these tests is the ankle brachial index (ABI), which is used to assess the perfusion of the lower-extremities. ABI, which is assessed using a Doppler ultrasound device, measures the ratio of tibial systolic artery pressure to brachial systolic artery pressure [3,4,10-13]. Thus, ABI can measure PAD of the lower-extremities [5,14].

As a lower extremity PAD diagnostic tool, ABI has been determined its sensitivity, and specificity compared with angiography, The specificity of the ABI was high, but sensitivity of the ABI was not as high as the specificity (Sensitivity 72%-95%, Specificity 99% -100%) [5]. Although PAD is an important health issue, it is not sufficiently recognized by the general population and by clinicians [13,15,16]. Early diagnosis is important, and an accurate diagnosis can usually made from a medical history, physical examination and measurement of ABI [17-19]. Despite its high prevalence, PAD goes undiagnosed by many family practitioners [12,19]. For example, about one-third to one-half of patients is asymptomatic. The common clinical symptoms of PAD are pain, cramp and hypokinesia in the extremities. These symptoms are induced by exercise, which increases the oxygen requirement of the extremities, but disappear with resting [10]. Symptoms therefore result from progressive ischemia, depending on stenosis and obliteration in the arterial system. Intermittent claudication (pains in leg muscles during walking) is the most frequent and earliest symptom [10,14]. The risk of intermittent claudication increases with age, serum cholesterol concentration, hypertension, smoking, diabetes and coronary artery disease, and is higher in men than in women [5]. Clinical findings supporting a diagnosis of PAD in the affected extremities include arterial color changes (e.g. pallor), decreased or absent peripheral pulses, numbness, weakness, coolness and hypokinesis, burning, deterioration in nail structure, hair loss, and wounds. Ulceration and gangrene of the lower extremities are the most serious findings, with more than one-third of patients requiring amputation of the affected extremity [10,14].

Ignorance of PAD by primary care physicians represents a barrier to the elimination of secondary risk factors for myocardial infarction and stroke [12,13,17]. Thus, it is important to increase PAD knowledge and awareness of primary family physicians, the first physicians usually consulted by patients. We therefore assessed the PAD prevalence rate and its relationship to age, sex and cardiovascular risk factors, in elderly individuals in Turkey.

## Methods

### Setting

We planned to assess elderly individuals residing at the highest-capacity Geriatric Care Center and Residential Home in Izmir, Turkey. During the pre-work stage, three family physicians working at this institution were trained about PAD diagnosis and ABI measurement by a cardiovascular surgery specialist.

### Participants

Three family physicians working full-time at a geriatric care center and residential home and trained in the diagnosis of PAD invited patients residing at the home to come to the polyclinic to participate in the study. All participants were more than 60 years old and they or a relative worked as a civil servant. In addition, all participants were able to pay to stay at the unit, and their socio-economic level was generally high. This unit has two part, one is residential home and the other one is geriatric intensive care center. Patients with sufficient self-care live in residential home. They move to geriatric intensive care center in case of insufficient self-care. Total number of 642 elders staying at the Residential Home, the study included 507 participants.

### Tools

Family physicians were instructed on the use of the Microdop 8 brand portable Doppler ultrasound device to be used in practice. ABI was first measured on a sample group of patient. We designed a data collection form, which included cardiovascular risk factors (age, sex, history of hypertension, diabetes, smoking status, anticoagulant use, hyperlipidemia), other chronic diseases (cerebral diseases, CAD, chronic obstructive pulmonary disease (COPD), renal diseases), PAD clinical findings, and ABI measurements.

### Data Collection

Data were recorded on the forms from records in health files and by asking participants. Information about the diagnosis and treatment of chronic diseases, as well as blood lipid profiles and use of anticoagulants, were obtained from patients records as all patients were followed by their family physicians. Before ABI measurements, patients were rested for a minimum of 10 minutes. Participants were told to lie on the examination couch in a supine position, and systolic blood pressures were measured in the right and left brachial artery, posterial tibial artery and dorsalis pedis artery using the portable Doppler Ultrasound device.

Patients with ABI < 0.90 (based on The American College of Cardiology and the American Heart Association guidelines) were preliminarily diagnosed with PAD and referred to the cardiovascular surgery polyclinic of the university for further assessment. These patients were subsequently treated, followed-up and examined by specialists. We planned to complete the study within three months.

Our research confirmed to the Helsinki Declaration and to local legislation, and was approved by the Ethics Committee of Ege University, Izmir.

### Statistical Analyses

This was a descriptive, observational, cross-sectional study. Factors analyzed included general characteristics, cardiovascular diseases, other chronic diseases, general risk factors, treatments and past cardiovascular operations. Patients with and without PAD were compared using SPSS software (SPSS for Windows, version 11.5; SPSS Inc, Chicago, IL), using descriptive statistics for frequencies, chi-square tests for comparisons of independent proportions, with Fisher's exact test where appropriate; and Student's t-tests for comparison of means of independent groups. All analyses were two-tailed, and a p value < 0.05 was defined as statistically significant.

## Results

### General characteristics of participants

Of the 507 residents of the residential home who agreed to participate in this study, 317 (62.5%) were women and 190 (37.5%) were men. Their mean age was 77.61 ± 6.93 years (range, 62-102 years), with 261 (51.5%) aged 75-84 years.

The most prevalent chronic disease in these participants was hypertension, present in 383 (75.5%), with 306 of the latter (79.7%) having blood pressures lower than 140/90 mmHg. Other frequently observed chronic diseases included CAD, hyperlipidemia, type 2 DM, cerebral disease and COPD. We found that 293 individuals (57.8%) were taking acetylsalicylic acid during the study period.

### Prevalence of PAD, risk factors and other chronic diseases

**Figure 1:**
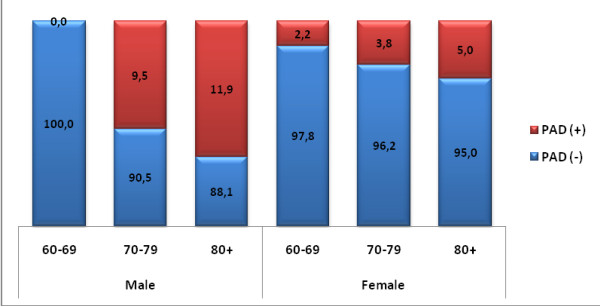
**Distribution of elderly PAD by gender and age**.

Of the 507 participants, 30 (5.9%) had an ABI < 0.90 and were referred to the cardiovascular surgery polyclinic with a diagnosis of PAD. Of these 30 patients, 17 (56.7%) were men, compared with 174 (36.5%) of those in the non-PAD group. Mean age was significantly higher in the PAD than in the non-PAD group (p = 0.010). Distribution of PAD patients by age groups and gender is shown in Figure 1.

We found that 27 of 30 (90.0%) patients in the PAD had hypertension, compared with 357 (74.8%) in the non-PAD group (p = 0.077). The frequency of type 2 DM was significantly higher in the PAD (12 patients, 40.0%) than in the non-PAD (111 patients, 23.3%) group (p = 0.048), as was the frequency of CAD (16 patients, 53.3% vs. 127 patients, 26.6%; p = 0.003). There were no significant differences found the rates of body mass index (BMI) between PAD and non-PAD groups. In addition, the rates of cerebral disease and COPD were higher in the PAD than in the non-PAD group.

Smoking during any period of life was significantly more frequent in the PAD than in the non-PAD group (15 patients, 30.0% vs. 131 patients, 27.5%; p = 0.012). Smoking history was therefore a risk factor for individuals not having chronic disease, whereas age was the only risk factor for atherosclerosis. In PAD group, two elders had CAD, one cerebrovascular event while the fourth elder had both diseases so that need to use clopidogrel. In the non-PAD group, 10 elders use clopidogrel because of CAD, and 7 elders use clopidogrel because of the cerebrovascular event. Chronic diseases, anticoagulant treatments, habits, and cardiologic operations in the PAD and non-PAD groups are shown in Table 1.

**Table 1:** Characteristics of Elderly According to Their PAD Status

Characteristics	PAD (-)	PAD (+)
Age in years, mean (SD)*	77.42 (6.96)	80.77 (5.69)

	n (%)	n (%)

Participants	477 (94.1)	30 (5.9)
Male Gender*	174 (36.5)	17 (56.7)
**Risk Factors**		
Hypertension	356 (74.6)	27 (90.0)
Type 2 DM *	111(23.3)	12 (40.0)
Hyperlipidemia	119 (25.0)	11 (36.7)
Smoking*	131 (27.5)	15 (50.0)
**BMI**		
Normal	185 (38.8)	11 (36.7)
Overweight	202 (42.3)	11 (36.7)
Obese	90 (18.9)	8 (26.6)
**Other Chronic Disease**		
Coronary artery disease*	127 (26.6)	16 (53.3)
Cerebral disease	81 (17.0)	6 (20.0)
Chronic obstructive pulmonary disease*	47 (9.9)	8 (26.7)
**Other clinical conditions**		
History of MI	29 (6.1)	3 (10.0)
Alcohol	49 (10.3)	7 (10.9)
**Cardiovascular surgery**	71 (14,9)	6 (20)
**Anticoagulant treatments**		
Acetyl salicylic acid	272 (57.3)	21 (70)
Clopidogrel*	17 (3.6)	4 (13.3)
Coumadin	19 (4.0)	2 (6.7)

SD: Standard deviation.

*p < 0.05

**Figure 2:**
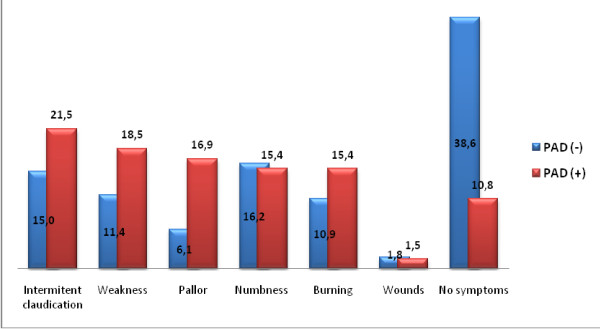
**Symptoms of the elderly**.

Intermittent claudication was present in 15 PAD patients (50%), comprising of 6 women (46.5%) and 9 men (52.9%). There were no findings in 7 patients (23%), including one with no relative risk factors other than age and male gender. Clinical findings of PAD patients are shown in Figure 2.

## Discussion

### Main findings

This study is the first extensive determination by primary health care providers of the prevalence of PAD and related risk factors in elderly Turkish individuals. To date, the epidemiology of PAD in Turkey had not been determined [20]. Assessments of PAD by primary health care services may result in a more efficient approach to PAD [4,12,20]. Because of the specificity of the ABI was high, potential false positive is very low. Thus it is very useful for finding the prevalence of PAD in this study population [5].

In assessing the demographic characteristics of the elderly participants in the study, we found that about two-thirds were women and the mean ages of both men and women were high. Of these individuals, 5.9% had ABI < 0.90 and were referred to the cardiovascular surgery polyclinic with the diagnosis of PAD.

### Discussion of the study findings under the light of the literature

Previous studies have shown that the prevalence of PAD is higher in men than in women and increases with age [5,7,21]. The mean age of our PAD group and the percentage of men were both higher than those of our non-PAD group, similar to previous findings.

Of our 30 PAD patients, 29 had atherosclerotic risk factors. These risk factors were more prevalent in our PAD than in our non-PAD group, with type 2 DM, COPD, smoking history, and CAD all being significantly higher in the PAD group. On the other hand obesity was similar between two groups. These results were expected, inasmuch as atherosclerosis is a significant risk factor for both PAD and CAD, and risk factors for atherosclerosis, including hypertension, smoking, diabetes and hyperlipidemia, are associated with PAD [4-6,11]. Only one of our PAD patients had no risk factors than advanced age and male gender.

We found that the rate of PAD among our participants was lower than expected for this age group. This low PAD rate may have been due to their relatively good health care and treatments. About 60% of participants were taking acetylsalicylic acid regularly. Moreover, about 70% of individuals with ABI < 0.90 PAD were taking anticoagulants. In general, anti-platelet agents are recommended for individuals with vascular diseases, with acetylsalicylic acid usually administered to individuals with CAD, cerebral diseases and PAD [4,10]. The participants in this study were residents at the highest capacity institution in Turkey, with full-time family physicians and a strong interdisciplinary team providing efficient primary health care service [22]. Our findings thus indicate that risk factors for PAD may be minimized by preventive health care services, which reduces risk factors, and by treatment for chronic diseases by family physicians and nurses, providing continuous patient care.

About one quarter of our PAD patients had been diagnosed before the study, with the remaining three quarters not previously diagnosed or treated for this condition. This low rate of diagnosis and treatment is likely due to the nature of this disease, which is rarely diagnosed early [8,12,19]. Even in countries with developed family medicine practices, more than half of PAD patients remain diagnosed, although 55% of these could be diagnosed by scanning [12]. While PAD has risk factors similar to those for atherosclerotic diseases, physicians and patients are relatively unaware of PAD, with awareness increasing only after the diagnosis of disease [16].

A study showing that one-fifth of individuals in the United Kingdom aged 65 to 75 years had evidence of PAD on clinical examination also found that only one quarter of individuals with PAD were symptomatic [18]. By contrast, about three quarters of our PAD patients were symptomatic. The higher rate of symptomatic patients in the present study may be due to the advanced age of our study group.

The incidence of intermittent claudication in PAD patients has been reported to depend on age and gender. Of individuals in Turkey diagnosed with PAD and assessed angiographically, 73.9% had intermittent claudication, with 90% being male [20]. Risk factors for intermittent claudication include male gender, increased age and smoking [5,8]. We found that 52.9% of the patients in our PAD group had intermittent claudication, with the rate being higher for men than for women. However, the incidence of intermittent claudication in our PAD patients was likely associated with age.

### Strengths and limitations

The most important limitation of this study was that the participants live in an institution. These participants are therefore at higher socio-economic and educational levels than the general population. In this geriatric care center and residential home, health services are provided by family physicians, including treatment for chronic diseases, thus reducing the incidence of risk factors for PAD.

### Implications to the study

PAD is a vascular disease frequently observed by primary care physicians in individuals with cardiovascular diseases such as cerebral diseases and CAD, with symptoms usually observed only in patients with advanced stage disease [10,21]. ABI is an important diagnostic tool that is easy to utilize in family medicine. Extensive epidemiological studies of the prevalence of PAD relative to medical history and ABI are necessary in Turkey.

## Conclusions

Generally, risk factors for PAD other than genetics can be modified and this leads to reduction in its incidence. Family physicians being aware of PAD may lower its risk factors, as well as diagnose it with ABI, and treat this condition and refer to specialists when needed. Coordination between primary care physicians and consultant cardiovascular surgeons is very important for the efficient diagnosis and treatment of PAD and for preventing complications. It is of critical importance to increase family physicians' awareness of PAD and to take into consideration PAD when diagnosing patients.

## Competing interests

The authors declare that they have no competing interests.

## Authors' contributions

NT participated in the design, coordination and analysis of the study. MB and TY participated in data collection and contributed in the coordination of the study. OK participated in the design and analysis of the study. All authors read and approved the final manuscript.

## Pre-publication history

The pre-publication history for this paper can be accessed here:

http://www.biomedcentral.com/1471-2296/12/96/prepub
